# Polysaccharides-Based Complex Particles’ Protective Role on the Stability and Bioactivity of Immobilized Curcumin

**DOI:** 10.3390/ijms22063075

**Published:** 2021-03-17

**Authors:** Camelia-Elena Iurciuc (Tincu), Leonard Ionuţ Atanase, Christine Jérôme, Vincent Sol, Patrick Martin, Marcel Popa, Lăcrămioara Ochiuz

**Affiliations:** 1 Department of Pharmaceutical Technology, Faculty of Pharmacy, Grigore T. Popa University of Medicine and Pharmacy, 16 University Street, 700115 Iaşi, Romania; camelia_tincu83@yahoo.com (C.-E.I.); ochiuzd@yahoo.com (L.O.); 2 Department of Natural and Synthetic Polymers, Faculty of Chemical Engineering and Protection of the Environment, Gheorghe Asachi Technical University, 73 Prof. Dr. Docent Dimitrie Mangeron Street, 700050 Iasi, Romania; 3 Department of Biomaterials, Faculty of Dental Medicine, Apollonia University, 11 Pacurari Street, 700355 Iasi, Romania; leonard.atanase@yahoo.com; 4 Center for Education and Research on Macromolecules, CESAM-RU, University of Liège, Allée du 6 août 13, 4000 Liège, Belgium; 5 Laboratoire PEIRENE EA 7500, University of Limoges, 87000 Limoges, France; vincent.sol@unilim.fr; 6 UniLaSalle, Unité Transformations & Agroressources, Université d’Artois, ULR7519, 62408 Béthune, France; patrick.martin@univ-artois.fr; 7 Academy of Romanian Scientists, 54 Splaiul Independentei Street, 050085 Bucharest, Romania

**Keywords:** polysaccharides particles, polymeric matrix, immobilized curcumin, pH and light stability, curcumin metal complexes, antioxidant activity, BSA adsorption

## Abstract

The curcumin degradation represents a significant limitation for its applications. The stability of free curcumin (FC) and immobilized curcumin in complex particles (ComPs) based on different polysaccharides was studied under the action of several factors. Ultraviolet-visible (UV-VIS) and Fourier-transform infrared (FTIR) spectroscopy proved the FC photodegradation and its role as a metal chelator: 82% of FC and between 26% and 39.79% of curcumin within the ComPs degraded after exposure for 28 days to natural light. The degradation half-life (t_1/2_) decreases for FC when the pH increases, from 6.8 h at pH = 3 to 2.1 h at pH = 9. For curcumin extracted from ComPs, t_1/2_ was constant (between 10 and 13 h) and depended on the sample’s composition. The total phenol (TPC) and total flavonoids (TFC) content values increased by 16% and 13%, respectively, for FC exposed to ultraviolet light at λ = 365 nm (UVA), whereas no significant change was observed for immobilized curcumin. Antioxidant activity expressed by IC_50_ (µmoles/mL) for FC exposed to UVA decreased by 29%, but curcumin within ComPs was not affected by the UVA. The bovine serum albumin (BSA) adsorption efficiency on the ComPs surface depends on the pH value and the cross-linking degree. ComPs have a protective role for the immobilized curcumin.

## 1. Introduction

Inflammatory bowel diseases (IBD) are characterized by chronic non-infectious inflammation of the gastrointestinal tract and include Crohn’s disease, ulcerative colitis, and colitis of undetermined cause [1,2]. The treatment and prevalence of IBD were described in a recent paper, which is the first part of the present study [3].

In recent years, polyphenols, such as curcumin, have gained significant attention in complementary and alternative treatment owing to the high-safety therapeutic dose (up to 12 g/day) and the wide range of beneficial pharmacological effects [4,5,6]. In vitro and in vivo research studies suggest that curcumin is a natural agent with positive influences on gastrointestinal health and function. Curcumin appears to influence the intestinal microbiota composition, modulates the intestinal barrier’s permeability, and reduces inflammation and oxidative stress in the gastrointestinal tract [5,7,8,9].

The solubilization of curcumin in the gastrointestinal tract fluids is relatively weak, which means that the polyphenol’s absorption by epithelial cells is inefficient [10]. The chemical instability and degradation of curcumin in alkaline pH in aqueous solution are well documented [11,12,13,14,15] and involve the hepta-dienone fragment’s rupture, leading to the active methylene group’s disappearance responsible for its antioxidant activity [16]. Trans-6-(4′-hydroxy-3′-methoxyphenyl)-2,4-dioxo-5-hexenal, ferulic aldehyde, ferulic acid, vanillin, vanillic acid, and other secondary products obtained from dimerization reactions were identified [17,18]. Supplementary Figure S1 shows the main curcumin degradation products. Previous research studies also show that if curcumin (in crystalline or solubilized form) is exposed to sunlight, it suffers a photodegradation determined by the chromophore groups’ presence [16,18,19,20,21,22]. The photodegradation products are similar to those identified after the curcumin degradation in alkaline solutions.

Curcumin has a good metal chelator activity, but its pharmacological properties could be reduced in the presence of metal ions [23]. It has been found that the cytotoxic effect was lower when curcumin complexes with Zn^2+^ were tested on prostate cancer cells compared to free curcumin (FC) and can induce DNA damage due to pro-oxidative activity [24].

Several formulations were prepared to increase bioavailability and achieve more efficient systemic circulation, better permeability, and resistance to metabolic processes, including nanoparticles, liposomes, micelles, nanofibers, and phospholipid complexes [25,26,27,28]. The local release of curcumin using pH-sensitive polymeric nanoparticles based on poly(lactic-*co*-glycolic acid) (PLGA) and Eudragit^®^ S100 in vitro and in vivo has been evaluated. Nanoparticles with immobilized curcumin significantly improved the drug’s permeability in Caco-2 cell cultures compared to the free curcumin (FC) in suspension and reduced the inflammation in the colon of mice with IBD. It should be noted that a higher accumulation of curcumin was observed in both types of mice, healthy and with IBD, when curcumin-loaded nanoparticles were administered, compared to the curcumin suspension administration [29,30].

Chitosan, i-carrageenan, and gellan were previously used to obtain different drug administration systems in the colon [31,32,33,34,35,36]. Still, few studies highlight the protective role of the polymer matrix for curcumin to the action of various environmental factors that can cause its degradation. Zheng et al. [37] studied curcumin’s stability encapsulated in both alginate- and chitosan-based hydrogel particles compared to FC in solution during storage in two different pH media (pH = 3 and 7) for 14 days. The authors found that the degradation of the immobilized curcumin in alginate particles was more intense, at both pH values, compared to curcumin encapsulated in chitosan particles. However, the FC degradation at pH = 7 in phosphate buffer solution (PBS) was more intense than encapsulated curcumin [37]. Core-shell nanoparticles based on biopolymers were obtained by self-assembly using a hydrophobic protein (zein) as a core and ionically cross-linked iota-carrageenan (ι-carrageenan) with low Ca^2+^ ions concentrations for the outer hydrophilic layer formation. The nanoparticles obtained can control the release of co-encapsulated active principles (curcumin and piperine) in the gastrointestinal conditions in vitro, and their bioavailability can be improved when they are administered by the oral route. It was also found that the antioxidant activity of curcumin and piperine co-encapsulated into nanoparticles increases due to the hydrophilic surface of the particles, which ensures better contact between the active principles and free radicals [38]. Previous research studies have presented that chitosan in contact with blood could lead to thrombosis and embolization [39]. When sulfonated groups (RSO3^–^) were included on the macromolecular chain, the positive charge determined by the protonated amino groups was reduced, and the plasma proteins’ adsorption, responsible for the early stages of the thrombogenic process, was lower [40,41].

This paper is a continuation of a previously published research article [3], which reports the obtaining and the physicochemical characterization of curcumin-loaded complex particles (ComPs) based on three polysaccharides (gellan/i-carrageenan/chitosan) obtained by ionic cross-linking and polyelectrolyte complexation. The designed ComPs contained chitosan nano/microparticles with immobilized curcumin, incorporated in a gellan or gellan mixed with i-carrageenan matrix, and were obtained by ionic cross-linking with bivalent ions and polyelectrolyte complexation. The ComPs were designed to allow efficient targeting and curcumin released into the colon. In the previous study [3], it was demonstrated that the obtained ComPs are gastro-resistant and could assure a controlled and sustained release of curcumin in the colon, being suitable for oral administration. The present study’s novelty and originality are to establish the protective role of the gastro-resistant ComPs based on polysaccharides for immobilized curcumin. Several factors that may affect curcumin’s stability (light stability, stability to the metal complexes formation, and stability in buffer solutions of different pH values) have been evaluated. Furthermore, the ultraviolet light’s influence, at λ = 365 nm (UVA), on the antioxidant activity and the total phenols (TPC) and flavonoids (TFC) content in FC for curcumin extracted from ComPs was also studied. Finally, the biocompatibility of ComPs was evaluated by determining the adsorption efficiency of bovine serum albumin (BSA) in three different pH media (pH = 2, 6.8, and 7.4), and the factors that may influence this behavior were discussed.

## 2. Results and Discussion

### 2.1. Photostability of Curcumin

It is well-known that curcumin is sensitive to light, being degraded (discolored) when exposed to natural light [42,43]. Starting from the hypothesis that the polymer matrix can protect curcumin from photodegradation, a comparative study was performed on FC and P2C, or P4C ComPs. It was considered that the P2C and P4C samples are the most relevant because the P2C sample has a medium cross-linking degree, resulting from the ionic gelation (the magnesium acetate concentration in the cross-linking bath was 2%), and the P4C sample contains the highest proportion of i-carrageenan in the polymer matrix (30%). As reported in the previous paper [3], the high amount of i-carrageenan induces the highest porosity in the formed particles, so we were interested in establishing how the particles’ porous morphology influences the natural light stability of immobilized curcumin.

**Figure 1:**
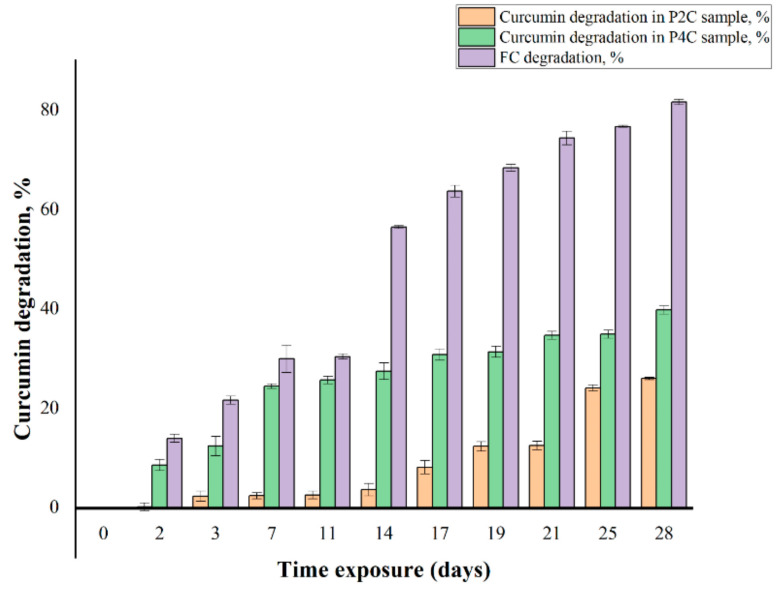
Curcumin degradation (%) of the free curcumin (FC) and the curcumin encapsulated in the P2C and P4C samples. All samples were exposed for 28 days to natural light and air.

It is well-known that the curcumin degradation at air and light can be evaluated spectrophotometrically. Thus, when FC was exposed to natural light and air, it was noted that the absorbance values at 425 nm decrease over time. The percentages (% *w/w*) of degraded curcumin, over time, at air and light of FC and curcumin-loaded samples are presented in Figure 1.

Figure 1 shows that the smallest amount of degraded curcumin after 28 days was observed for the P2C sample. Slightly higher values can be noticed for curcumin encapsulated in P4C samples, whereas the FC was highly degraded. Since curcumin was incorporated first in the chitosan matrix, and then these microparticles were included in the gellan or gellan/i-carrageenan matrix, it is obvious why the stability of curcumin-loaded in the ComPs was higher than that of FC. The slight difference between these two samples, P2C and P4C, can be explained by the presence of 30% i-carrageenan in the latter one, which induces a higher porosity to the matrix, thus facilitating the diffusion of air into the polymer network, which has a degrading action on curcumin. It has been found that the combined effect of air and light is more harmful than that of light alone [42]. The Student’s T statistical test shows that the differences between FC’s degradation and curcumin included in the P2C and P4C samples at natural light and air have statistical significance, at *p* < 0.05. Under these conditions, the order of the curcumin complex components’ photochemical stability, as reported by Lee et al. [42], was curcumin > demethoxycurcumin > bisdemethoxycurcumin.

From the ultraviolet-visible (UV-VIS) spectra presented in Supplementary Figure S2, recorded at different light exposure durations, it was found that the absorbance value decreases over time. These UV spectra demonstrate that a more pronounced degradation of FC occurs after 14 days.

**Figure 2:**
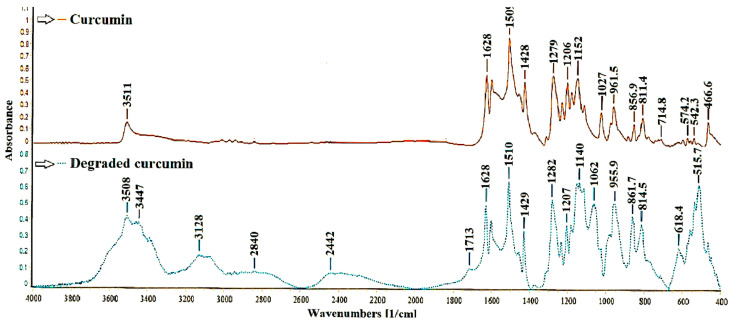
Fourier-transform infrared (FTIR) spectrum for degraded curcumin in the presence of light and air for 28 days compared to the FTIR spectrum of non-degraded curcumin.

The Fourier-transform infrared (FTIR) spectrum of curcumin photodegradation after 28 days of exposure to air and light is shown in Figure 2. It should be noted that although the literature mentions [42] the possibility of photodegradation of curcumin when exposed to light, no FTIR spectra are shown to confirm this effect.

The characteristic curcumin bands appear in both spectra, but there are also some differences, especially at 2300–3600 cm^−1^, respectively 400–900 cm^−1^. Generally, the bands from this region are assigned to the phenolic group ʋ_OH_. However, it could be noted that in the degraded curcumin spectrum, the number of absorption bands attributed to this group is higher, shifted, and intensified. The presence of new absorption bands at 2442, 2840, and 3128 cm^–1^ can also be observed, which can be attributed to the COOH group, and the new absorption peak from 3447 and 3508 cm^–1^ can be assigned to the OH group from a potential degradation product, namely ferulic acid or trans-6-(4′-hydroxy-3′-methoxyphenyl)-2,4-dioxo-5-hexenal.

The absorption bands between 1600 and 1650 cm^–1^ corresponding to aromatic rings and C=C/C=O conjugated are found in the non-degraded curcumin spectrum and the one of degraded curcumin. The degraded curcumin spectrum shows the presence of a distinct peak, at a lower intensity absorption band at 1713 cm^–1^, which is assigned to a carbonyl group (C=O vibration bands appear in the range 1700–1800 cm^–1^) and may indicate the presence of vanillin or ferulic aldehyde.

Other apparent differences between spectra occur in the 1000–1300 cm^–1^ region, with the degraded curcumin spectrum bands being considerably more intense than those of FC, even though they are found in both samples. It is particularly noteworthy that the absorption bands from 1140 and 1062 cm^–1^ of the degraded curcumin spectrum are much more intense. These peaks, attributable to the C-O stretching vibrations, are shifted more than in the non-degraded curcumin spectrum. They may occur due to the four degradation products’ formation, as mentioned earlier (ferulic acid, feruloyl methane, ferulic aldehyde, and vanillin). Stronger absorption bands are observed in the degraded curcumin spectrum in the 400–900 cm^–1^ region, assigned to the presence of the -C = C- bond (namely, the absorption peak from 814.5 cm^–1^ can be attributed to the alkene groups trisubstituted). These bands have significant importance in identifying potential degradation products (ferulic acid, feruloyl methane, ferulic aldehyde). These FTIR results suggest that curcumin was degraded in the presence of light and air.

### 2.2. Stability of Curcumin at Complexation with Metal Ions

Curcumin forms stable complexes with most known metal ions due to the unsaturated α, β-diketo group, an excellent chelating agent. Moreover, it was considered a bidentate monobasic ligand that could form stable complexes with almost all metals and non-metals at a stoichiometric ratio of 2:1 (curcumin/metallic ions) [16,44]. Recent studies have shown that the hydroxyl group’s deprotonation from the keto-enol group results in a β-diketonate form, which strongly chelates with Cu^2+^ or Zn^2+^ ions [45].

**Figure 3:**
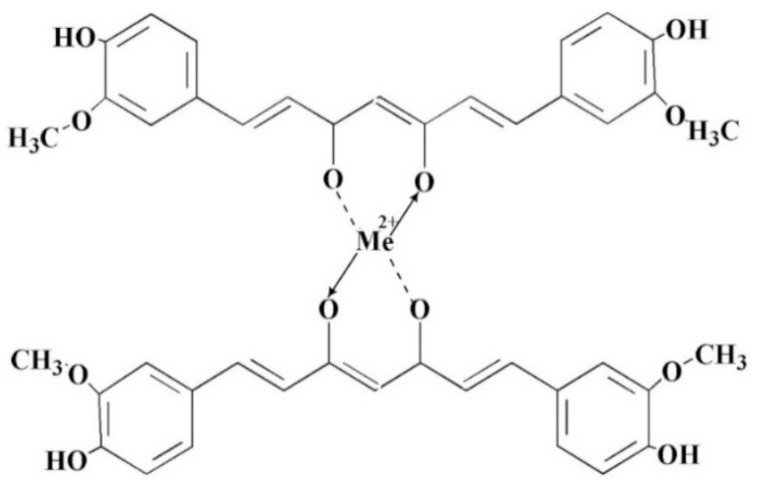
The theoretical structure of the curcumin/metal ions complex.

The structure of this complex is illustrated in Figure 3.

**Figure 4:**
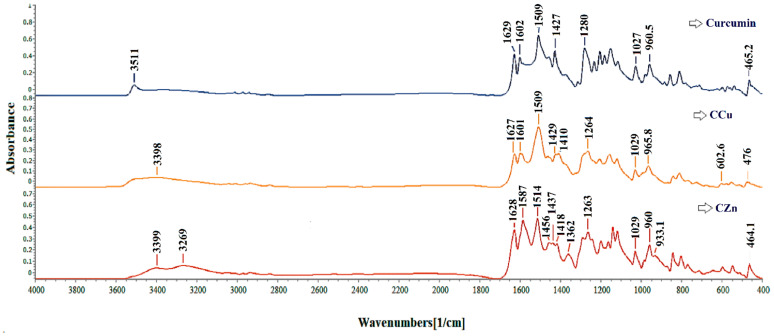
FTIR spectrum for free curcumin (FC) compared with the FTIR spectrum for the curcumin complexes with copper (CCu) and zinc (CZn) ions.

This section studied the influence of the ComPs on the curcumin stability at complexation with metal ions. Therefore, the FC was complexed with Cu^2+^ and Zn^2+^ ions, obtained from the corresponding sulfates in solution, and the P2C ComPs were immersed in the same Zn^2+^, and Cu^2+^ solution to determine if the stability of curcumin loaded in the polysaccharide-based matrices could be improved by avoiding the curcumin complexation with metal ions. For FC and curcumin complexed with Cu^2+^ and Zn^2+^ ions, the FTIR spectra are given in Figure 4.

Compared with the FC spectrum, its specific absorption bands are shifted in the spectrum of metal complexes of curcumin with Cu^2+^ and Zn^2+^ ions, indicating an interaction of the metal ions with the -OH groups. The wavelength band of 3511 cm^–1^, attributed to the enol band (-OH) from the FC spectrum, appears shifted in the spectra’s curcumin metal complexes. New absorption bands are observed in the metal complexes spectrum, at 3398 cm^–1^ for the curcumin complexes with Cu^2+^ or 3399 and 3269 cm^–1^, respectively, in the curcumin complexed with Zn^2+^ ions spectrum. It is known that the curcumin molecule can exist both in enolic form and in the form of diketone, the latter being able to complex with the metals. The complex of curcumin with the Zn^2+^ ions, formed by β-diketone, was previously identified by mass spectrometry [46,47].

The less intense absorption band at about 1602 cm^–1^ in the curcumin spectrum, attributed to C=O and C=C group, shifts and widens at 1600 and 1587 cm^–1^ in the metal complexes’ spectra. These results prove that the metal ion complexation occurs through the β-diketone fragment but does not exclude the possibility of interaction with phenolic groups.

The highest intensity absorption band is observed at 1509 cm^–1^, and it can be attributed to several groups, such as C=O, δ _CC=O_, and δ _CCC_ [48]. The metal complexes’ absorption bands are shifted to 1514 cm^–1^ in the Zn^2+^ complexes’ spectrum. The absorption bands in the 1450–1300 cm^–1^ region cannot be attributed to a single vibration, but their shifts in curcumin’s metal complexes’ spectra can be observed compared with the FC spectrum. Thus, the peak at 1427 cm^–1^, attributed to the δ_C-O_ enol band from the FC spectrum [49], is split into two small bands, 1429 and 1410 cm^–1^, in the curcumin complexes with Cu^2+^ spectrum. In the curcumin complexes with Zn^2+^ spectrum, the peaks are found at 1437 and 1418 cm^–1^. Also, in the spectrum of curcumin metal complexes with Zn^2+^ ions, the absorption band’s intensification at 1362 cm^–1^ from the curcumin spectrum can be attributed to the CO-CH_3_ structure, δ_CH_. Moreover, the intense absorption band that appears at 1280 cm^–1^, generally attributed to C-O (phenol) or C-O-CH_3_ in FC, is shifted to 1264 cm^–1^ for Cu^2+^ complexes and at 1263 cm^–1^ for Zn^2+^ complexes, indicating some interaction with metal ions.

Finally, the absorption band at 1027 cm^–1^ attributed to the CH=CH group in the FC spectrum is shifted to 1029 cm^–1^, and the absorption band from 960.5 cm^–1^ appears at 965.8 cm^–1^ in the metal complexes’ spectrum of curcumin with Cu^2+^ ions. For metal complexes of curcumin with Zn^2+^ ions, the 960.5 cm^–1^ absorption band is split into two peaks, one of which is more pronounced at 960 cm^–1^ and the other with a lower intensity at about 948 cm^–1^. The literature states that these changes in the adsorption bands from 960.5 cm^–1^ in the curcumin spectrum can be attributed to Me-O vibrations [50]. In the curcumin spectrum, the absorption band from 465.2 cm^–1^ can be attributed to the MeO structure [51] and appears shifted in the spectrum of curcumin metal complexes with Cu^2+^ and Zn^2+^ ions at 476 and 464.1 cm^–1^, respectively. These results demonstrate curcumin’s ability to form complexes with ions of transition metals, namely Cu^2+^ and Zn^2+^.

It is well-known that the UV wavelength at which the absorbance of curcumin/metal ions complexes has a maximum value is different from the one specific to FC [52,53]. Complexes of curcumin with copper and zinc ions have been reported in previous studies [44,54], and it was stated that metal complexes are solubilized only in highly polar solvents [10]. Previous studies report that the wavelength of curcumin dissolved in dimethyl sulfoxide (DMSO) at which the absorbance has a maximum value is equal to 434 nm [18].

**Figure 5:**
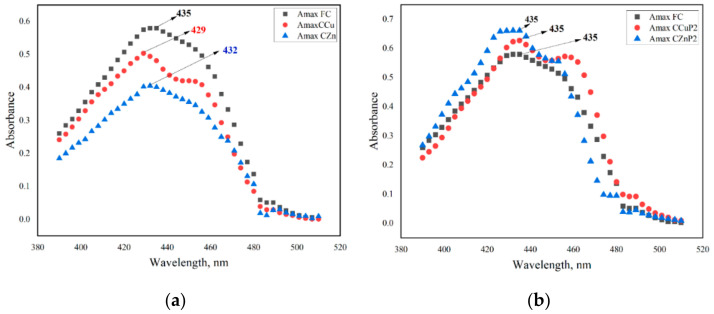
Ultraviolet (UV) spectra for FC, FC complexes with Cu^2+^ and Zn^2+^ ions (**a**) and for curcumin extracted from the P2C sample, which was previously immersed for 4 h in copper and zinc sulfate solutions (**b**).

By scanning multiple wavelengths between 390 and 510 nm, it was found, in this study, that the maximum absorbance value for FC dissolved in DMSO appears at a wavelength of 435 nm and that this value was slightly different for metal complexes. Figure 5 shows these results.

**Figure 6:**
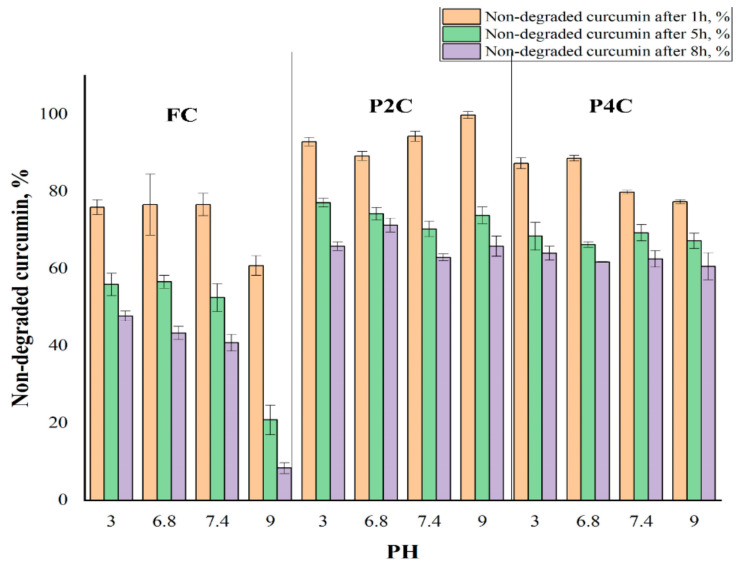
Variation of non-degraded degraded curcumin in time at different pH values for free curcumin (FC) and curcumin immobilized P2C and P4C samples.

Curcumin/metal ions complexes dissolved in DMSO have a maximum absorption band at different wavelengths, 429 and 432 nm, for complexes formed with Cu^2+^ and Zn^2+^ (Figure 6) than in the FC UV spectrum (435 nm). The shift of the FC absorption peak and shoulders’ appearance in the various complexes depends on the metal ion nature [53].

Changes in the absorption band for curcumin/metal ions complexes can be correlated with forming a new lower energy state due to the charge transfer by the transition of electrons from curcumin, which is considered ligand for metal ions. The delocalization of the electron from the orbital layer *p* from the ligand curcumin fragment, behaving as a donor, to the positively charged metal active sites having a free orbital layer *d*, which act as acceptors, leads to the formation of a smaller charge transfer band [22,55]. Zn^2+^ ion, having occupied the orbitals *d*, exhibits fewer interactions than the Cu^2+^ ions, for which these orbitals are free, which explains the expansion of the absorption band for the zinc ions [22].

Curcumin from P2C samples extracted in DMSO (after the maintaining of samples in zinc and copper solutions for 4 h) did not form metal complexes, the wavelength at which the absorbance of extracted curcumin from ComPs was maximum, being equal to that of FC, as illustrated in Figure 5b. Consequently, it can be admitted that the polymer matrix protects curcumin, and it can be expected that its pharmacological effects were not diminished. The results obtained are consistent with other previous studies, showing that curcumin immobilized in liposomes does not form metal complexes [24].

### 2.3. Curcumin Stability at Different pH Values

Curcumin is subjected to chemical degradation in aqueous solutions, and deterioration becomes more intense with increasing pH values.

Therefore, it was of interest to determine if curcumin’s encapsulation in polysaccharide-based ComPs prevents its degradation in buffer solutions with different pH. P2C and P4C samples were selected for this study. The P2C sample contains the curcumin-loaded chitosan microparticles in a gellan matrix, and the P4C sample contains the curcumin-loaded chitosan microparticles incorporated in a gellan mixed with a 30% i-carrageenan matrix. These samples were chosen because they were considered representative for this determination. The stability of curcumin encapsulated in these samples in different pH buffer solutions was compared to the one of FC. The degradation half-life in different pH buffers of the curcumin extracted from the P2C and P4C samples was assessed to determine the curcumin stability and bioavailability. The results concerning the value of the degradation rate constant and the half-life at degradation (t_1/2_) are provided in Figure 6 and Table 1 (Supplementary Table S1 presents the results obtained for all the samples for 8 h).

Figure 6 presents the percentage of non-degraded curcumin (FC and curcumin encapsulated in P2C and P4C samples) in solutions with different pH values and exposure times. The degradation rate constant and half-life at degradation for FC and curcumin extracted from P2C and P4C ComPs in different pH solutions are presented in Table 1. The results are given as mean value ± standard deviation (SD).

It is found that the FC degradation in time in buffer solutions with different pH was much more pronounced than the degradation of curcumin encapsulated in P2C and P4C samples, demonstrating the polymer matrix’s protective role. The curcumin degradation depends on the pH values and is intensified as it increases. Thus, after 8 h, it can be observed that the FC degradation was over 90% at pH = 9, while the degradation of curcumin encapsulated in P2C and P4C ComPs at the same pH value was 34.2% and 39.4%, respectively. The FC degradation was more intense in buffer solution at pH = 9 than the degradation in other solutions of different pH. It can be caused by subsequent hydrolysis of the formed secondary products, leading to the transformation of diferuloylmethane and ferulic acid into vanillin or acetone, which have no antioxidant activity [17]. Curcumin immobilized in particles was protected by the polymer matrix.

Previous research studies [56,57,58,59] and the results obtained in the first part of this article [3] show that the matrix porosity increases when the carrageenan amount increases in the polymer systems.

**Figure 7:**
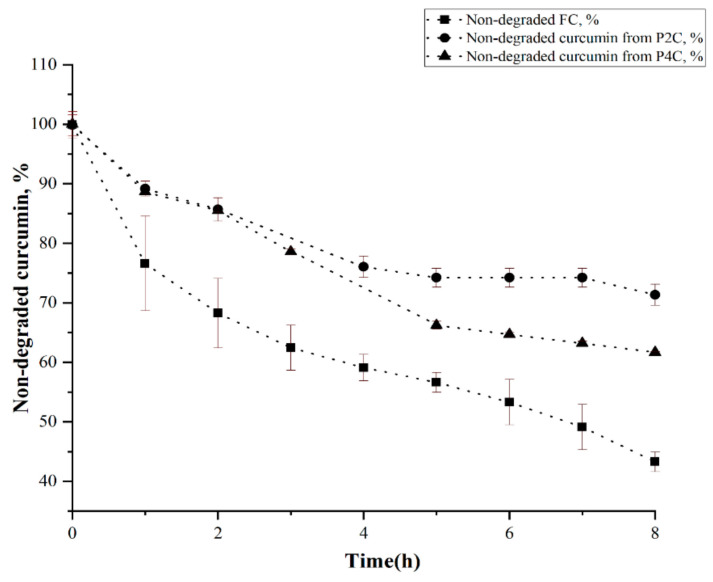
Variation in time in the amount of undegraded curcumin at pH 6.8 for free curcumin and curcumin extracted from P2C ComPs or P4C ComPs.

The degradation of curcumin immobilized in particles containing i-carrageenan (P4C) is slightly higher than curcumin immobilized in the matrix based only on gellan (P2C) due to a more pronounced porosity induced by i-carrageenan. The Student’s T statistical test shows that the differences recorded between non-degraded FC values and non-degraded curcumin values included in the P2C sample have statistical significance, where the *p* parameter’s value is lower than 0.05. The differences recorded between the amount of non-degraded FC and the amounts of non-degraded curcumin included in the P4C sample have statistical significance only at pH = 9, where *p* parameters are lower than 0.05. The difference between the degradation in time of curcumin included in the P2C ComPs and the degradation in time of curcumin included in P4C samples was not statistically significant. The value of the *p* parameter was higher than 0.05. The degradation of curcumin follows first-order kinetics, as shown in Figure 7.

Table 1 shows that FC was more stable in acidic pH (e.g., at pH = 3, t_1/2_ = 6.8 h), and its stability decreases as the pH value increases (at pH = 9, t_1/2_ = 2.3 h). The curcumin degradation mechanism was described in other studies [17,21,60]. More than 90% of FC decomposes rapidly after 8 h in buffer solution at pH = 9 because, as we mentioned earlier, the main degradation product at this pH is vanillin [20].

Previous research results have shown that curcumin from the curcuminoid mixture had better stability at pH = 7 than pure curcumin. Curcumin used in this study is part of a combination of curcuminoids, and FC degradation at pH = 7.4 was not so intense compared to other studies, in which degradation of pure curcumin in different pH buffer solutions was investigated [10,61].

For FC, the maximum half-life (t_1/2_) found by Vareed et al. [62] was 6.77 ± 0.83 h, being very close to the t_1/2_ value obtained for FC in an acidic medium (pH = 3). Moreover, previous research showed that the t_1/2_ determined at pH = 8 was equal to 1.5 min [14]. There is a possibility that the difference between the t_1/2_ values depends on the solution preparation procedure. Vareed et al. [62] used a concentration of 2% of methanol compared to the 1% concentration used by Naksuriya et al. [63] in a mixture with the buffer solution. Also, the use of a high concentration of curcumin, such as 37 μg/mL, may lead to curcumin precipitation, and consequently, to a more critical degradation [14,63].

The pH stability test was also performed for curcumin encapsulated in ComPs. The results showed that the polysaccharide matrix of the ComPs protects curcumin, and the degradation was avoided, both at acidic and basic pH values. Moreover, the degradation of the curcumin extracted from the P2C or P4C sample could be avoided because the buffer solutions need to diffuse first into the gellan matrix and then into curcumin-loaded chitosan microparticles.

Table 1 shows that the rate constant for FC at which degradation occurred at different pH values increases when the pH values increase while t_1/2_ was reduced. The degradation rate constant (k) for curcumin encapsulated in ComPs has a lower value than FC, and its value was almost constant regardless of the buffer solution pH used. This value was about 30% higher for the curcumin immobilized in the P4C sample than the one immobilized in the P2C sample. Thus, it can be concluded that t_1/2_ for curcumin encapsulated in the P2C and P4C ComPs was almost constant. Its value was higher than t_1/2_ for FC regardless of the solution pH in which the ComPs were immersed. The t_1/2_ value was 30% lower for curcumin within the P4C particles than the t_1/2_ for curcumin within the P2C sample. This effect may be due to the presence of i-carrageenan in the composition of P4C ComPs, which can lead to increased porosity and a lower cross-linking degree in the hydrogel particles, causing an intensified diffusion of the solution inside the ComPs and, thus, partial degradation of curcumin can occur. The t_1/2_ in buffer solutions of different pH values for curcumin encapsulated in the P2C sample was approximately 13 h at pH = 3, pH = 6.8, pH = 7.4, and t_1/2_ = 12 h at pH = 9. The t_1/2_ for curcumin encapsulated in the P4C sample was over 10 h in pH = 3, pH = 6.8, pH = 7.4, and it was smaller at pH = 9 (9.7 h).

**Figure 8:**
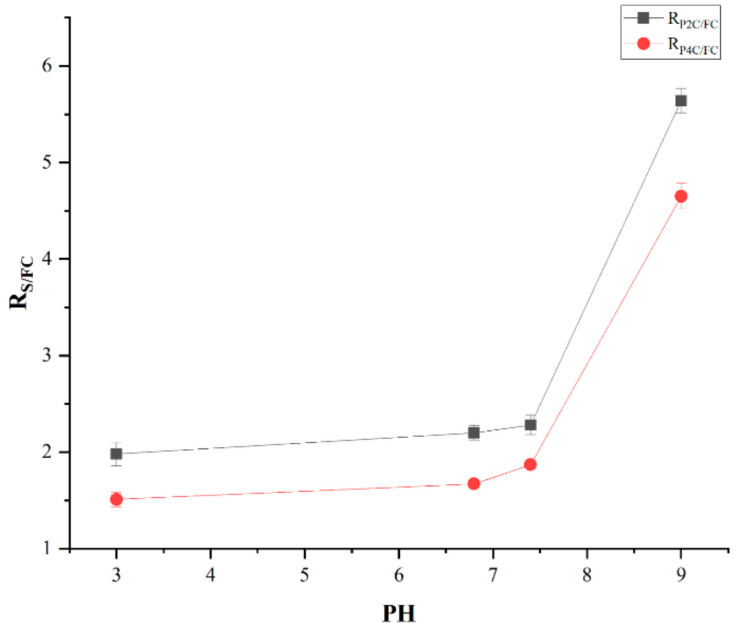
R_S/FC_ variation in function of pH values of the buffer solutions.

Moreover, the t_1/2_ for curcumin encapsulated in the P2C and P4C samples was higher than the one of FC, meaning that the curcumin stability in different pH solutions increases by its encapsulation. The slight decrease in t_1/2_ value for curcumin encapsulated in the P2C and P4C samples at pH = 9 can be caused by the fact that the ComPs contain gellan or gellan mixed with i-carrageenan. The basic pH induces the formation of carboxylate anions from acidic groups that did not participate in cross-linking with Mg^2+^ ions, causing electrostatic repulsions between polysaccharide chains, determining the polymer network relaxation, and the buffer solution diffusion within the ComPs can be facilitated. Figure 8 illustrates the curcumin degradation half-life (t_1/2_) ratio (noted R_S/FC_) at different pH values between the t_1/2_ for degradation of curcumin encapsulated in the P2C or P4C samples and t_1/2_ for FC. S represents the t_1/2_ at degradation for curcumin encapsulated in the P2C or P4C samples, and FC represents the t_1/2_ for FC degradation at different pH values.

It was found that at pH values in the acidic domain, even if the curcumin degradation increased and depended on the pH value, for both FC and immobilized curcumin, the ratio R_S/FC_, which suggests how effectively the polymer matrix protects curcumin, was kept practically constant. It is very interesting that at pH in the alkaline range, the matrix’s protection was much more effective (over 5.6 times in the case of the P2C sample, and respectively almost 4.5 times in the case of the P4C ComPs). When the samples were compared, it was found that the P4C matrix offers lower protection than the P2C matrix, which can be explained by the fact that, on the one hand, it has more functional groups that did not participate in the ionic cross-linking with Mg^2+^ or electrostatic interaction with amino groups from chitosan, and on the other hand, the higher proportion of i-carrageenan gives the matrix porosity, both effects leading to a more intense diffusion of the alkaline medium in the matrix respectively, to the immobilized curcumin, which it was degraded more intensely.

These results show that curcumin encapsulated in ComPs based on polysaccharides was protected against the factors that could affect its stability and bioavailability.

### 2.4. Determination of Total Phenol (TPC) and Flavonoid (TFC) Content of Curcumin

The TPC or TFC was determined for FC, UV-irradiated FC, and curcumin extracted from UV-irradiated and non-irradiated P2C, P4C, and P5C samples. The samples were UV-irradiated before the curcumin extraction in methanol. After encapsulation, the TPC and TFC of curcumin were analyzed to determine if the matrix has a protective role for curcumin. Polyphenols are sensitive in various environments during food processing and storage. Degradation of natural antioxidants may prevent the effectiveness of their therapeutic effects and avoid the use of these antioxidants in food/nutraceutical and pharmaceutical applications [64].

UV radiation represents a new approach for sterilizing biodegradable polymer matrices [65]. A UVA sterilization system (λ = 320–400 nm) was also designed for potable water, and it has been shown that irradiation with UVA for approximately 30 min can destroy non-pathogenic and pathogenic bacteria [66]. Plants have physiological, protective responses to harmful UV radiation. These responses include the synthesis of flavonoids, hydroxycinnamic acids, and their related compounds.

**Figure 9:**
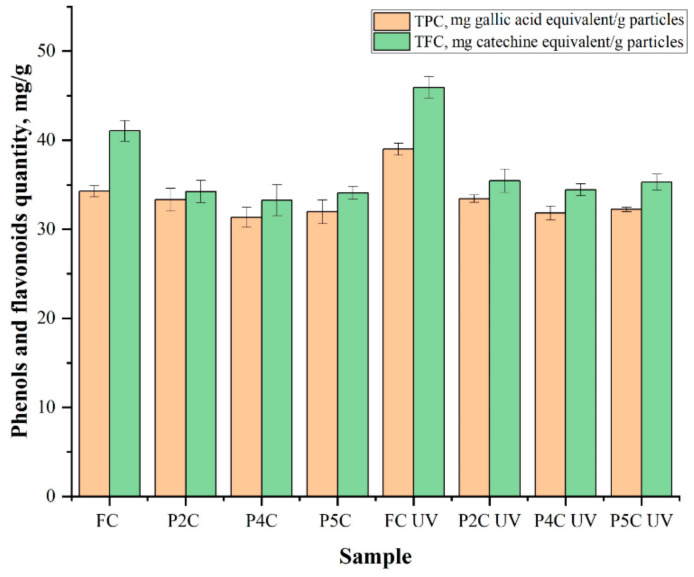
The total content of phenols (orange) and flavonoids (green) in free curcumin (FC) and the curcumin extracted from the P2C, P4C, and P5C samples that were irradiated and non-irradiated with UVA. Results were expressed as mean value ± standard deviation (SD). The samples exposed to UVA light were noted with P2CUV, P4CUV, or P5CUV and FCUV.

In this study, FC and curcumin immobilized in P2C, P4C, and P5C samples were exposed to ultraviolet light (UVA) at λ = 365 nm for 30 min using a UV lamp (UVPUVLMS-38 EL Series UV Lamp, Thermo Fisher Scientific, Cambridge, UK), for sterilization, and established if the polymeric matrix protects curcumin from photodegradation. Figure 9 and Supplementary Table S2 present the results obtained for the TPCs and TFCs analysis, expressed as mg of gallic acid equivalent (mg GAE)/g particles respectively, in mg of catechin equivalents (mg CE)/g particles. The theoretical total quantity of curcumin that can be immobilized in one gram of particles was 80 mg, and TPC and TFC determined from non-irradiated FC (TPC = 34.27 ± 0.63 mg gallic acid equivalent/g particles, TFC = 41.05 ± 1.17 mg catechins equivalent/g particles) or UV-irradiated (TPC = 39 ± 0.67 mg gallic acid equivalent/g particles, TFC = 45.91 ± 1.21 mg catechins equivalent/g particles) were considered as control (the TPC or TFC in the samples should not exceed this value because not all curcumin was immobilized). The equivalent values of the TPC and TFC in FC were obtained by Sepahpour et al. [67].

Figure 9 and Supplementary Table S2 show that UVA radiation affects the TPC or TFC in the FC. The TPC or TFC in FC exposed to UVA light increased compared to FC. Lee et al. [68] reported an increase in the total phenols and flavonoid content in irradiated plants. Such an increase in the total phenols and flavonoid content can be caused by the degradation of phenolic compounds with larger molecules into degradation compounds with smaller molecules or by releasing phenolic compounds from glycosidic components, as explained before by Harrison and Were [69,70]. The TPC and TFC from curcumin immobilized in P2C, P4C, and P5C samples were not significantly affected by UV radiation compared to those from FC. For FC exposed to UVA light, TPC increased by 13.8%, and TFC increased by 11.8% compared to the TFC or TPC in non-irradiated FC because curcumin could be degraded when it is exposed to UV light, and as previously mentioned by Lee et al. [68], also more small molecules of phenolic compounds could be formed and the TPC and TFC increase. The TPC increases for curcumin extracted from samples exposed to UVA light compared to the TPC of curcumin extracted from samples not exposed to UVA as follows: by 0.3% for curcumin extracted from the P2CUV sample, by 1.4% for curcumin extracted from the P4CUV sample, and by 0.8% in the case of curcumin extracted from the P5CUV sample. TFC also increases for curcumin extracted from samples exposed to UVA light compared to curcumin extracted from samples not exposed to UV light as follows: by about 3.4% for curcumin extracted from P2CUV samples and 3.5% for P4CUV and P5CUV samples.

The Student’s T statistical test shows that the differences between the TPC or TFC for UV-irradiated and non-irradiated FC or curcumin extracted from the P2C, P4C, and P5C ComPs have statistical significance in most cases. The exceptions were the TPC differences for P2C and FCUV samples or TPC for curcumin extracted from non-irradiated P2C, P4C, and P5C samples, and curcumin extracted from the same samples that were UV-irradiated, which are not statistically significant. Likewise, the differences in the TFC recorded between curcumin included in the P2C and P4C non-irradiated samples and the same UV-irradiated samples have no statistical significance, *p* > 0.05.

TPC and TFC for curcumin extracted from P4CUV and P5CUV samples increased slightly compared to curcumin extracted from the P2CUV sample. The TPC and TFC from curcumin immobilized in the polysaccharides ComPs depend on the immobilization efficiency and increase when the porosity decreases. It can be stated that the polymer matrix, in which curcumin was included, protects it quite effectively against the stress caused by UV radiation, and the results show that such formulations can be sterilized by UV irradiation.

### 2.5. Antioxidant Activity

**Figure 10:**
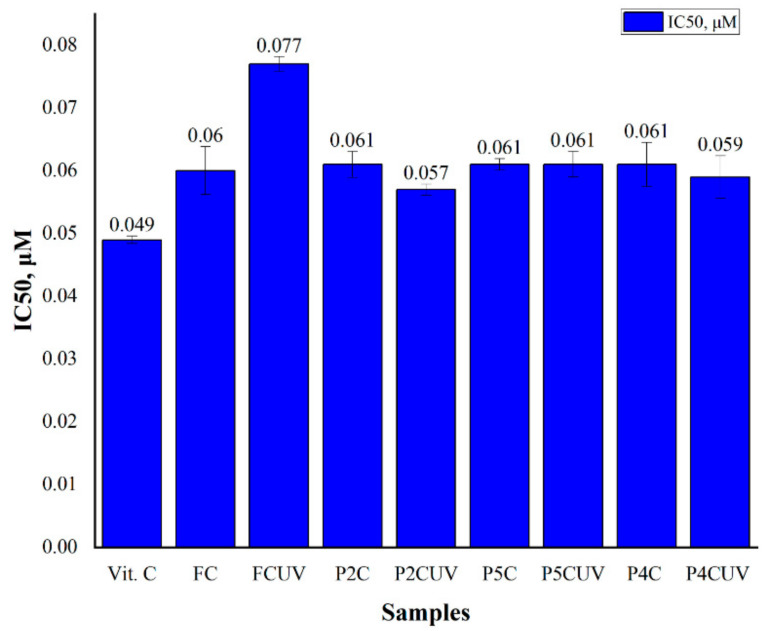
IC_50_ values on DPPH radical scavenging assay for free curcumin (FC) and curcumin extracted from P2C, P4C, and P5C samples before and after exposure to UV light for 30 min at 365 nm.

The FC and curcumin’s antioxidant activity extracted from P2C, P4C, and P5C samples were evaluated using the 2,2-diphenyl-1-picrylhydrazyl (DPPH) assay. The ComPs were exposed to UVA light for 30 min, and the curcumin from the ComPs was extracted into ethanol. In order to determine the inhibition percentage, I%, curcumin solutions of different concentrations (between 10 and 50 µg/mL) were used. IC_50_ was calculated from the graph inhibition percentage (I% vs. concentration and represents the sample’s concentration that can capture 50% of the free radicals in DPPH. IC50 value (µmoles/mL) represents the inhibition concentration of curcumin at which the DPPH radicals were scavenged by 50%, and it was calculated based on the curve interpolation of linear regression analysis. From these results, IC_50_ was determined for both curcumins extracted from UV-irradiated and non-irradiated ComPs. Figure 10 shows the IC_50_ values (µM) for ascorbic acid, FC, and curcumin extracted from P2C, P4C, and P5C samples (non-irradiated and irradiated with UVA light for 30 min, λ = 365 nm). When the IC_50_ values are low, curcumin’s antioxidant character is high.

From Figure 10, it can be noted that IC_50_ values for FC, curcumin extracted from UV irradiated or non-irradiated P2C, P4C, and P5C samples are close, which means that immobilized curcumin retains its antioxidant properties, and the polymeric matrices had a protective role. For FC exposed to UVA light, it appears that the IC_50_ value increased by 29% compared to that of FC, which was not exposed to UVA light (from 0.06 to 0.077 µM). The results are consistent with those obtained for the TPC and TFC determination in FC, where TPC increased by 16% and TFC increased by 13% after UVA exposure. The literature mentions that curcumin (powder) bioactivity was changed when it was exposed to UVB light, and 50% of curcumin was degraded after 24 h of exposure. After exposure to UVA, the FC antioxidant activity can be determined by the degradation products that contain the active hydroxyl methoxyphenyl group essential for the curcumin antioxidant activity [71].

The results obtained for P2C, P4C, and P5C samples before and after UVA exposure are also consistent with the samples’ TPC, and the values of IC_50_ obtained for every sample were very close. Once again, it is verified that the polymer matrix of P2C particles offers better protection of the immobilized curcumin, having the lowest IC_50_ value. The result is consistent with those previously presented.

For all samples exposed to UVA, the curcumin extracted from ComPs retains its antioxidant activity, with the IC_50_ value being close to that of FC. The differences between IC_50_ values for FC and those of curcumin extracted from irradiated or non-irradiated ComPs were not statistically significant, demonstrating that the matrix of gellan or gellan mixed with different amounts of i-carrageenan protects curcumin from UV radiation. This protection could be determined by the fact that two polymeric layers defend the polyphenol against UVA degradation. Curcumin is protected first by the chitosan micro/nanoparticles in which it was immobilized and then by the gellan or gellan/i-carrageenan matrix in which these chitosan micro/nanoparticles have been included. The TPC and TFC after UV irradiation of FC were higher compared to UV non-irradiated FC. Still, they did not determine the FC’s enhanced antioxidant activity, as claimed by previous research [72,73]. The increase of TPC and TFC after UVA exposure may result from curcumin degradation product formation with lower molecular weight, determining the decrease of antioxidant activity. The results show that the curcumin bioactivity, including its antioxidant properties, can be altered by UV radiation, and these effects must be considered during the sterilization of the release systems.

### 2.6. Proteins’ Adsorption

The protein adsorption study on the biopolymer particles’ surface is essential in biomedical applications, both in vitro and in vivo [74]. In this study, bovine serum albumin (BSA) was chosen as a model protein because it is chemically similar to human serum albumin (HSA) (the protein with the highest blood plasma concentration). Different proteins and enzymes should be adsorbed on the particles’ surface with immobilized drugs in the gastrointestinal tract [73].

**Figure 11:**
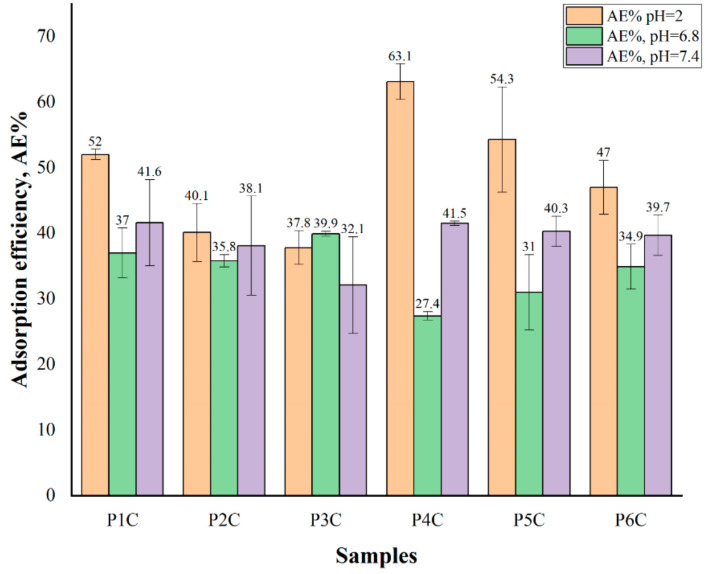
Bovine serum albumin (BSA) adsorption efficiency (AE%) for all samples obtained after simultaneous placement of the ComPs at pH = 2 (blue), pH = 6.8 (red), and pH = 7.4 (green). Results are expressed as mean values ± SD.

The interactions between protein molecules and polymer particles can be classified as hydrophobic interactions, ionic (or electrostatic) bonds, hydrogen bonds, and van der Waals interactions [73]. The results obtained for the BSA adsorption efficiency on the surface of the P1C, P2C, P3C, P4C, P5C, and P6C samples are presented in Figure 11.

From the obtained results, it was found that the BSA adsorption depends on the electrostatic interactions, the swelling degree, and the ComPs composition. At pH = 2, the BSA adsorption increases as the cross-linking degree decreases, and the i-carrageenan quantity within the ComPs increases. At this pH, the BSA amine groups are protonated, and they can interact with the carboxylic groups of gellan or the sulfate groups of i-carrageenan on the surface of the ComPs that did not participate in the cross-linking reaction.

As the i-carrageenan amount increases (contains two sulfate groups in each structural unit) in the particles’ composition, the samples’ porosity increases [3], and the number of free functional groups in the polymer matrix also increases.

At pH = 6.8, it was observed that BSA adsorption efficiency values in ComPs had lower values than at pH = 2. At pH = 6.8, the BSA’s amino groups were not protonated, and no electrostatic interactions occurred, resulting in a lower BSA adsorption on the surface of the ComPs. It could be noted for the P3C sample, with a higher cross-linking degree, that the BSA adsorption efficiency value was slightly higher than for P1C or P2C samples, which have a lower cross-linking degree. Also, the BSA adsorption efficiency values at pH = 6.8 tend to decrease with the increasing quantity of the i-carrageenan in the complex particle’s composition.

In the previous research article [3], it was demonstrated that, at pH = 2, hydrogen bonds could be formed because the pKa value for gellan is about 3.5, and the pKa value for i-carrageenan is about 2.6 [75,76]. The number of hydrogen bonds within ComPs can increase when the cross-linking degree decreases, and the hydrogen bonds can be cleaved in time [3].

There is the possibility that at pH = 6.8, the protein adsorption also depends on the hydrogen bonds previously formed at pH = 2 and not only on the cross-linking degree. An explanation for the results obtained would be that the hydrogen bonds previously formed at pH = 2 within the ComPs were not cleaved immediately after the immersion of samples in BSA solution at pH = 6.8. These hydrogen bonds could be cleaved in time after a period in which the ComPs adsorb a sufficient amount of BSA solution in PBS at pH = 6.8. Thus, for the P3C sample, the BSA adsorption was improved because the number of hydrogen bonds formed was lower than in the other ComPs. As the i-carrageenan concentration within P4C, P5C, and P6C samples increases, the functional group’s number also increases, leading to a higher number of hydrogen bonds previously formed at pH = 2 within the ComPs. Consequently, the BSA adsorption efficiency at pH = 6.8 increases when the quantity of i-carrageenan in the sample decreases.

The swelling degree has a maximum value at pH = 7.4 and increases when the cross-linking degree decreases [3]. Compared with the protein adsorption at pH = 6.8, a higher amount of BSA solution could diffuse within the polysaccharide particles at pH = 7.4 because the hydrogen bonds, initially formed at pH = 2, were cleaved. At pH = 7.4, there were not electrostatic interactions between the amino and carboxylate groups of gellan or sulfate groups of i-carrageenan because the isoelectric point of BSA is at pH = 4.5, and the amino groups were not protonated at this pH value. Consequently, the BSA adsorption efficiency was lower than at pH = 2. Moreover, curcumin at pH = 7.4 becomes more soluble. At this pH, hydrogen bonds can be formed between the hydroxyl groups of curcumin and the amino groups of albumin, leading to improved BSA adsorption efficiency compared to the one obtained at pH = 6.8.

The protein adsorption on the surface of ComPs in the gastrointestinal tract depends on the pH value, the cross-linking degree, and the interactions between BSA and the constituent polysaccharides.

## 3. Materials and Methods

### 3.1. Materials

Complex particles based on gellan/carrageenan/chitosan with curcumin immobilized were previously obtained [3]. Curcumin powder (extracted from *Curcuma Longa*), Tween 20, bovine serum albumin (BSA), dimethyl sulfoxide (DMSO), hydrochloric acid (37%), 2,2-diphenyl-1-picrylhydrazyl (DPPH), Folin-Ciocalteu reactive, double tartrate of sodium, and potassium were purchased from Sigma Aldrich, Hamburg, Germany Sodium nitrite (NaNO_2_), Aluminum chloride (AlCl_3_)_,_ Sodium carbonate (Na_2_CO_3_), Sodium hydroxide (NaOH), Copper sulfate (CuSO_4_ × 5H_2_O), ethanol were provided by the Chemical Company (Iași, Romania).

### 3.2. Preparation of the Complex Particles

The ComPs can improve the curcumin administration orally, increase the curcumin bioavailability in the colon, help achieve the therapeutic concentration, and the active substance could be released at the colon’s target site. Their preparation method was based on the formation of polyelectrolyte complexes between three polysaccharides and involves two steps to prepare the curcumin-loaded particles, as described in a previous study [3]. Supplementary Figure S3 presents the schematic representation of the preparation process of obtaining the polysaccharides’ ComPs with immobilized curcumin, and it was also given in the previously published article [3]. In order to determine if the ComPs protect curcumin from external factors, tests were required to assess the immobilized curcumin’s stability compared to FC. The sample’s code and its composition are presented in Table 2.

### 3.3. Characterization Methods

#### 3.3.1. FTIR Spectroscopy

FTIR spectra were obtained for FC and degraded curcumin (in solid-state, as powder) under the light and air (Section 2.1), and as well as for the metal complexes of curcumin with zinc and copper (in solid-state, as powder) (Section 2.2), which were recorded using the potassium bromide (KBr) pellet method. The spectra were recorded on a Bruker FTIR Spectrometer (VERTEX 70) over a wave number range of 400–4000 cm^–1^ at a resolution of 2 cm^–1^.

#### 3.3.2. Photostability of Curcumin

Light stability of FC and encapsulated curcumin was determined photocolorimetrically by using a BOECO UV-VIS model S22 spectrophotometer (Hamburg, Germany). It is known from the literature that the absorbance decreases at the same wavelength (425 nm) if a polyphenol was degraded [77]. The test was performed in the presence of natural light and air inside the laboratory of the Faculty of Chemical Engineering and Environmental Protection, “Gheorghe Asachi” Technical University, Iasi (Latitude 47°9′6.2136″ N, Longitude 27°35’16.4904″ E). The samples’ exposure was made in natural light in June 2020 (between June 2 and 29, so in summer) to verify whether keeping the samples in these conditions is possible, without degrading encapsulated curcumin, or the extent to which curcumin degradation occurs. The length of the day (light duration) in June was, on average, 15.5 h. The samples were placed in uncovered Petri dishes placed behind the glass window inside of the laboratory. The place where samples were maintained allowed sunlight and air to act on the particles containing immobilized curcumin. The laboratory temperature did not exceed 25 °C. The whole period was practically sunny, with no clouds. The analyzed P2C and P4C dry ComPs (0.45 g from each sample) and FC (1 g) were exposed to air and natural light for 28 days in uncovered Petri dishes. Several particles were taken from each sample at different time intervals, weighed, and the encapsulated curcumin was extracted with ethanol (10 mL). The absorbance was measured at 425 nm, and based on the calibration curve in ethanol, the amount of non-degraded curcumin was calculated. The results were expressed as a percent (%) of degraded curcumin, and it was calculated with Equation (1) as follows:

(1) $$\mathrm{Degraded}\;\mathrm{curcumin}\;\%\;=\frac{\;\mathrm{Amount}\;\mathrm{of}\;\mathrm{degraded}\;\mathrm{curcumin}}{\mathrm{Initial}\;\mathrm{curcumin}\;\mathrm{amount}\;\mathrm{within}\;\mathrm{one}\;g\;\mathrm{of}\;\mathrm{particles}}\times \;\;100$$

The equation of the calibration curve was: y = 0.1951x, R^2^ = 0.9996. All determinations were made in triplicate, and the results were presented as mean value ± standard deviation.

#### 3.3.3. Curcumin Stability at Complexation with Metal Ions

The obtaining of metal complexes of curcumin with Zn^2+^ and Cu^2+^ was reported in different research studies [78,79]. Previous studies have shown that the wavelength at which the absorption had a maximum value for the curcumin/metal ions complexes was different from that of FC [53]. Therefore, the wavelength at which UV absorbance has a maximum value was determined for FC, for curcumin/metal ions complexes, and for curcumin extracted from the ComPs that were stored before in metal ions solutions. The metal ions selected for this study were Cu^2+^ and Zn^2+^ (aqueous sulfate solutions). Firstly, FC/metal ion complexes, at a molar ratio of 2:1 (2.7×10^–4^ moles Zn^2+^ or Cu^2+^ and 5.4 × 10^–4^ moles of FC) were prepared, as follows: curcumin was dissolved in 10 mL of ethanol, and the copper or zinc sulfate amounts were dissolved in 5 mL of distilled water. The Cu^2+^ or Zn^2+^ aqueous solutions were added dropwise to the alcoholic curcumin solution. After 4 h, under stirring, at 50 °C, a brown precipitate was observed for samples complexed with Cu^2+^ and a red one for samples complexed with Zn^2+^. These precipitates were filtered, washed with bi-distilled water, and dried at 50 °C in an oven, then, 5 mg of each of these complexes were dissolved in 10 mL DMSO, and the absorbance was determined by scanning multiple wavelengths ranging from 390 to 510 nm. A known amount of ComPs containing 5 mg of curcumin was maintained in the aqueous solution of copper or zinc sulfate (2.7 × 10^–4^ moles, 15 mL) under gentle stirring for 4 h at room temperature. The ComPs were separated by filtration and washed two times with bi-distilled water. The sample was then immersed in 10 mL DMSO to extract the curcumin from ComPs, and after extraction, the ComPs were separated by decantation. The wavelength at which the absorbance has a maximum value was established for the curcumin extracted in DMSO from the P2C by scanning wavelengths ranging from 390 to 510 nm samples, and a single absorption band corresponding to FC was observed (λ = 435 nm).

#### 3.3.4. Curcumin Stability at Different pH Values

The procedure for determining FC stability to pH was adapted from Kumavat et al. [80]. Briefly, 10 mg of curcumin was dissolved in 100 mL of ethanol, and from this stock solution, 1 mL was taken in order to prepare a 25 mL solution with a curcumin concentration of 4 μg/mL and different pHs values, such as 3, 6.8, 7.4, and 9. The samples were maintained, at 37 °C, in the dark, in closed containers, for 8 h. The curcumin concentration from these solutions was determined spectrophotometrically based on the calibration curve, at λ = 425 nm, every hour for 8 h. For the curcumin’s pH stability determination encapsulated in the ComPs, the P2C and P4C samples were selected. A quantity of dry curcumin-loaded particles was used to obtain the same curcumin concentration of 4 μg/mL as in FC solutions. The samples were immersed in buffer solutions (25 mL) with different pH values, as previously mentioned, being maintained at 37 °C in the oven and protected from interaction with light or air. For each analyzed sample, eight samples were prepared in quoted volumetric flasks (25 mL) at each pH value. The content of each sample was analyzed, using a spectrophotometer, every hour for 8 h, as follows: the buffer solution in which the samples were immersed was removed by simple decantation, and then an accurately measured volume of ethanol (10 mL) was added to extract the curcumin from the ComPs for 1 h at ambient temperature. The concentration of curcumin in the extract was determined spectrophotometrically based on the calibration curve of curcumin in ethanol, at λ = 425 nm. It was expressed as a percent of non-degraded curcumin in buffer solutions of different pH values’ function of time. Based on these results, a straight line was plotted between the logarithm of non-degraded curcumin concentration and time, and both the degradation rate constant, k, and the degradation half-life, t_1/2_, were calculated [81,82,83]. Therefore, the degradation half-life, t_1/2_, represents the time when the initial concentration decreases by a half. The relationship (2) and (3) below suggests that it depends on the initial concentration and constant reaction rate:

(2) t12=A02k

where: [A]_0_ represents the initial concentration, k is the degradation rate constant, and t_1/2_ represents the half-life at the degradation.

For the reactions governed by first-order kinetics, the slope decreases continuously over time. t_1/2_ for the first-order reactions depends only on the degradation rate constant, k, and not on the substance’s initial concentration. The t_1/2_ equation, in this case, is:

All determinations were made in triplicate, and the results are expressed as the mean value ± standard deviation (SD).

(3) t12  = ln 2k ∼0.693k

#### 3.3.5. Determination of the Total Phenols (TPC) and Flavonoids (TFC) Content

TPC and TFC were determined for FC and curcumin extracted from ComPs before and after exposure to ultraviolet light (UVA) at 365 nm for 30 min using a UVPUVLMS-38 EL Series UV Lamp (Thermo Fisher Scientific, Cambridge, UK) Determination of the Total Phenols Content (TPC)

The method was adapted with minor modifications from References [84,85]. TPC was quantified using the Folin-Ciocalteu assay. Solutions of different FC concentrations and curcumin extracted from ComPs exposed or not to UV light at 365 nm for 30 min in methanol were prepared: 1 mL from each solution was dissolved in 5 mL of Folin-Ciocalteu reagent. The mixture was kept for 5 min at room temperature, and then 4 mL of Na_2_CO_3_ solution (7.5% *w/v*) was added. The solutions were vortexed and maintained at room temperature for 1 h. The absorbance was measured at a wavelength of 765 nm. Solutions of various gallic acid concentrations (used as standard) in methanol were prepared, and the calibration curve was plotted. All experiments were performed in triplicate, and the Student’s T statistical test was used to determine the statistical significance of the obtained results. The equation of the gallic acid calibration curve was: y = 0.0171x, R^2^ = 0.9836.

##### Determination of the Total Flavonoids Content (TFC)

With some modification, the method was adapted from References [86,87]. Solutions of different FC concentrations and curcumin extracted from the ComPs obtained in methanol were prepared: 1 mL of each solution of different concentrations was diluted with 4 mL of bi-distilled water, and then 150 μL of 5% sodium nitrite solution (NaNO_2_) was added. After 5 min, 300 μL of 10% (*w/v*) AlCl_3_ solution was added, and the solution was maintained at room temperature for 5 min before adding 2 mL of 1 N NaOH solution. The reaction mixture thus obtained was diluted by adding 550 μL of distilled H_2_O. The solutions were vortexed for 30 s, and the absorbance of the solutions was measured at 510 nm after 15 min. Catechin was used as a standard, and the calibration curve was plotted to determine the TFC. All experiments were performed in triplicate, and the Student’s T statistical test was used to determine the statistical significance of the obtained results. The equation of the catechin calibration curve was: y = 0.0043x, R^2^ = 0.995.

#### 3.3.6. Antioxidant Activity Determination

With some modifications, the work method was described before by Choi et al. [88]. The stock solution of curcumin was prepared by dissolving 25 mg of curcumin in 50 mL of ethanol. In order to determine the antioxidant activity of curcumin, several dilutions were performed from the stock solution. The final curcumin solution concentrations were between 10 and 50 μg/mL, and 2 mL of each solution was added to the tubes. Over the tubes with curcumin solutions, 2 mL of 0.1 mM DPPH solution was added. Samples thus prepared were vortexed for 20 s. The samples’ absorbance was read after 40 min using a UV spectrophotometer at a wavelength of 517 nm. Ascorbic acid was used as a standard. Absorbance values were converted in antioxidant activity percent (inhibition percent of free radicals from DPPH) using the Equation (4):

(4) $$I\left[\%\right]\;=\;100\;-\;\left[(A_{s_{}}-A_{b})\;\;\times \;\;\frac{100}{A_{c}}\right]$$

IC_50_ was calculated from the graph I% vs. concentration and represented the sample’s concentration that can capture 50% of the free radicals in DPPH. A_s_ represents the absorbance value of the solutions of different curcumin concentrations containing 2 mL of 0.2 M DPPH and 2 mL sample. As blank (A_b_) was prepared by adding 2 mL of ethanol and 2 mL of different curcumin solution concentrations in the test tubes. The blank absorbance was measured for each concentration separately to consider the TFC in samples. The control solution, A_c_, was prepared using the DPPH solution (2 mL) and ethanol (2 mL). The determinations were performed spectrophotometrically at 37 °C after the samples were maintained in the dark for 40 min. A part of the ComPs was UV-irradiated for 30 min at λ = 365 nm, and the curcumin immobilized in ComPs was extracted into ethanol. Solutions of different concentrations were used to determine the inhibition percentage, and based on the results obtained, IC_50_ was determined for both curcumins extracted from UV-irradiated ComPs and curcumin extracted from non-irradiated ComPs. All determinations were performed in triplicate.

#### 3.3.7. Determination of Protein Adsorption

The protein adsorption was studied in three different pH environments: pH = 2 (solution prepared using 10 mM NaCl and 0.1 N HCl, simulating the pH of the gastric environment), pH = 6.8 (PBS—similar to the intestinal fluid), and pH = 7.4 (PBS—similar for blood and colonic fluids). The protein used as a model was bovine serum albumin (BSA), and three solutions with different pH were prepared at a concentration of 4 mg/mL. In order to determine the proteins’ adsorption under conditions that simulate those of the gastrointestinal tract, 50 mg of curcumin-loaded ComPs was placed successively for 2 h (representing the particles’ residence time in the gastric medium) in 1 mL of BSA solution at pH = 2, then for 3 h (representing the particles’ residence time in the intestinal medium) in 1 mL of BSA solution at pH = 6.8, and finally, for 4 h (representing the particles’ residence time in the colonic medium) in 1 mL of BSA solution, at pH = 7.4. After each determination, the particles were separated by filtration and washed with bi-distilled water. The washing waters were mixed with the BSA solutions of different pH, in which the ComPs were immersed, and the volumes of the BSA solutions combined with the washing waters were determined. Using the Lowry method [89], the amount of BSA from each solution of different pH was determined spectrophotometrically at λ = 660 nm. The BSA amount adsorbed by the curcumin-loaded ComPs was determined through the difference between the initial amount of BSA and the BSA amount measured in solutions of different pH values. The recipients containing ComPs suspended in the BSA solutions were closed and maintained at 37 °C in the dark in an oven. The results were expressed by the adsorption efficiency (AE%) in every solution of different pH, and it is described by Equation (5):

(5) $$A_{E_{}}\%\;=\frac{\;\mathrm{Total}\;\mathrm{amount}\;\mathrm{of}\;\mathrm{BSA}\;\mathrm{absorbed}}{\mathrm{The}\;\mathrm{initial}\;\mathrm{amount}\;\mathrm{of}\;\mathrm{BSA}\;\mathrm{in}\;1\;\mathrm{ml}\;\mathrm{of}\;\mathrm{solution}}\;\;\times \;\;100$$

The BSA calibration curve equation was: y = 0.0008x, R^2^ = 0.9966. All determinations were made in triplicate, and the results are presented as mean value ± SD.

#### 3.3.8. Statistical Analysis

Results are presented as the mean ± SD. The Student’s T-test was used for assessing unequal variance. A two-tailed *p*-value less than 0.05 was considered significant.

## 4. Conclusions

The influence of some factors on FC stability and curcumin encapsulated in ComPs based on gellan/i-carrageenan/chitosan was investigated. It was observed that the degradation of FC was intensified after 28 days of exposure to natural light and air compared with the curcumin within ComPs. The degradation at natural light or in different pH solutions was intensified for curcumin encapsulated in the ComPs, containing 30% i-carrageenan due to the polymer matrix’s increased porosity. The pH degradation study in buffer solutions of FC and curcumin incorporated in ComPs showed that curcumin’s chemical degradation was intensified when the pH value increased. The degradation half-life, t_1/2_, decreases when the pH value increases for FC, while the t_1/2_ for encapsulated curcumin in the P2C or P4C samples remains almost constant and increases at approximately 13 h, or respectively, 10 h. Studies on curcumin complexation in the presence of Zn^2+^ and Cu^2+^ ions showed that encapsulated curcumin did not form metal complexes, and the maximum absorbance value change for Zn^2+^ and Cu^2+^ complexes of curcumin at the same wavelength. The antioxidant activity decreased for FC exposed to UV. Curcumin extracted from irradiated or non-irradiated ComPs retained its antioxidant activity, and the IC_50_ values were very close to that of the FC that was not exposed to UVA light. The results obtained for antioxidant activity are consistent with the results obtained for TPC and TFC determination. The protein adsorption efficiency depends on the pH value, the electrostatic interactions, the cross-linking degree, and the interactions between BSA and polysaccharides. UVA light could sterilize the obtained ComPs with curcumin encapsulated before biological tests. ComPs have a protective role for curcumin, allowing it to overcome the gastric barrier and to be absorbed more efficiently into the colon. To demonstrate the advantages of these curcumin-loaded polysaccharide-based particles, ex vivo and in vivo tests will be carried out in a future study.

## Figures and Tables

**Table 1:** The degradation rate constant and t_1/2_ at degradation for FC and curcumin encapsulated in P2C and P4C ComPs in different pH solutions.

Sample	PH	k	t_1/2_ (h)
FC	3	0.10 ± 0.005	6.8 ± 0.3
	6.8	0.11 ± 0.006	6.2 ± 0.3
	7.4	0.12 ± 0.001	5.7 ± 0.05
	9	0.32 ±0.025	2.1 ± 0.1
P2C	3	0.051 ± 0.003	13.5 ± 0.8
	6.8	0.05 ± 0.002	13.6 ± 0.5
	7.4	0.053 ± 0.002	13.1± 0.6
	9	0.059 ± 0.001	11.8 ± 0.3
P4C	3	0.067± 0.003	10.35 ± 0.5
	6.8	0.067 ± 0.001	10.34 ± 0.1
	7.4	0.065 ± 0.001	10.7 ± 0.2
	9	0.071 ±0.002	9.7 ± 0.3

**Table 2:** The polymer matrix composition and the encapsulation efficiencies.

Sample *	Gellan (%)	i-Carrageenan (%)	Magnesium Acetate Solutions Concentrations (%), 100 mL	Encapsulation Efficiency (%)
P1C	100	0	1	97.25
P2C			2	91.5
P3C			3	87.23
P4C	70	30	2	85.71
P5C	80	20	2	90.4
P6C	90	10	2	94.2

* In the polymer matrix, curcumin-loaded chitosan micro/nanoparticles were encapsulated with a percentage of 11%.

## Data Availability

The data presented in this study are available on request from the corresponding author.

## Supplementary Materials

The following are available online at https://www.mdpi.com/1422-0067/22/6/3075/s1, Figure S1: The degradation products of curcumin, Figure S2: UV-VIS spectra recorded for FC degradation in the presence of air and light, Figure S3: Schematic representation of the preparation process of obtaining the polysaccharides’ ComPs with immobilized curcumin. Table S1: Variation of non-degraded curcumin (%) in time, in buffer solutions with different pH values, for the analyzed samples, Table S2: The TPC or TFC determined from free curcumin (FC) and curcumin extracted from P2C, P4C, and P5C samples irradiated or not irradiated with UVA. The results are expressed as mean values ± SD.
