# Supplementary material for: Polysaccharides-Based Complex Particles’ Protective Role on the Stability and Bioactivity of Immobilized Curcumin
Source: Int J Mol Sci. 2021 Mar 17;22(6):3075. doi: 10.3390/ijms22063075 (PMC8002829; doi:10.3390/ijms22063075)
Supplement: Supplementary file 1 [file ijms-22-03075-s001.pdf]

International Journal of
Molecular Sciences

MDPI

Supplementary Materials

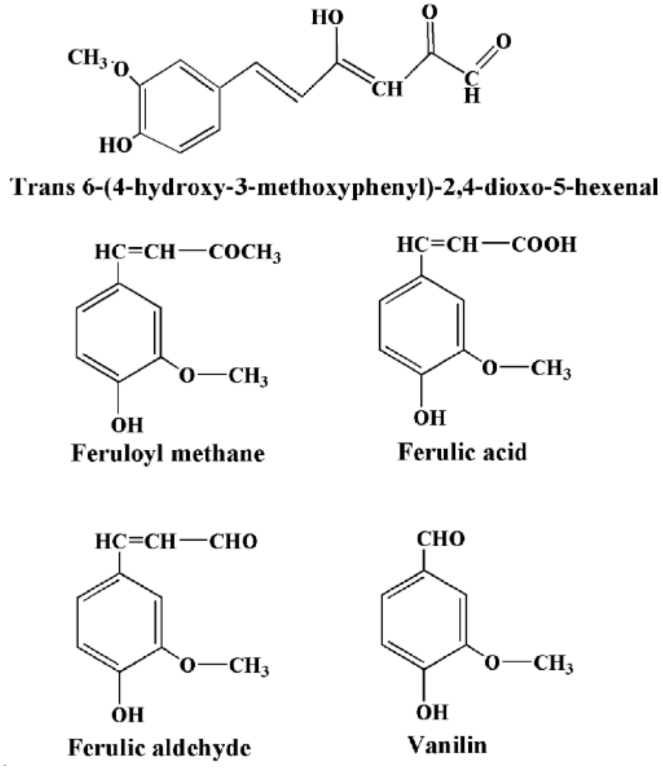

Figure S1. The degradation products of curcumin.

Day 0.

Day 2

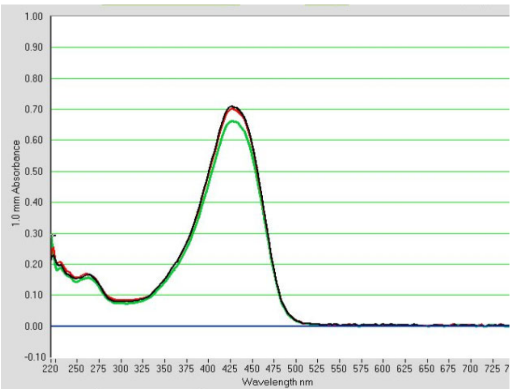

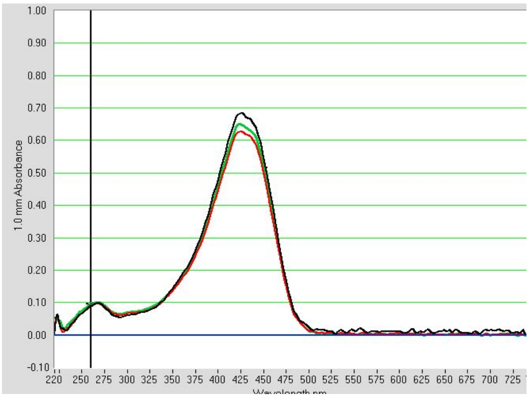

Day 4

Day 15

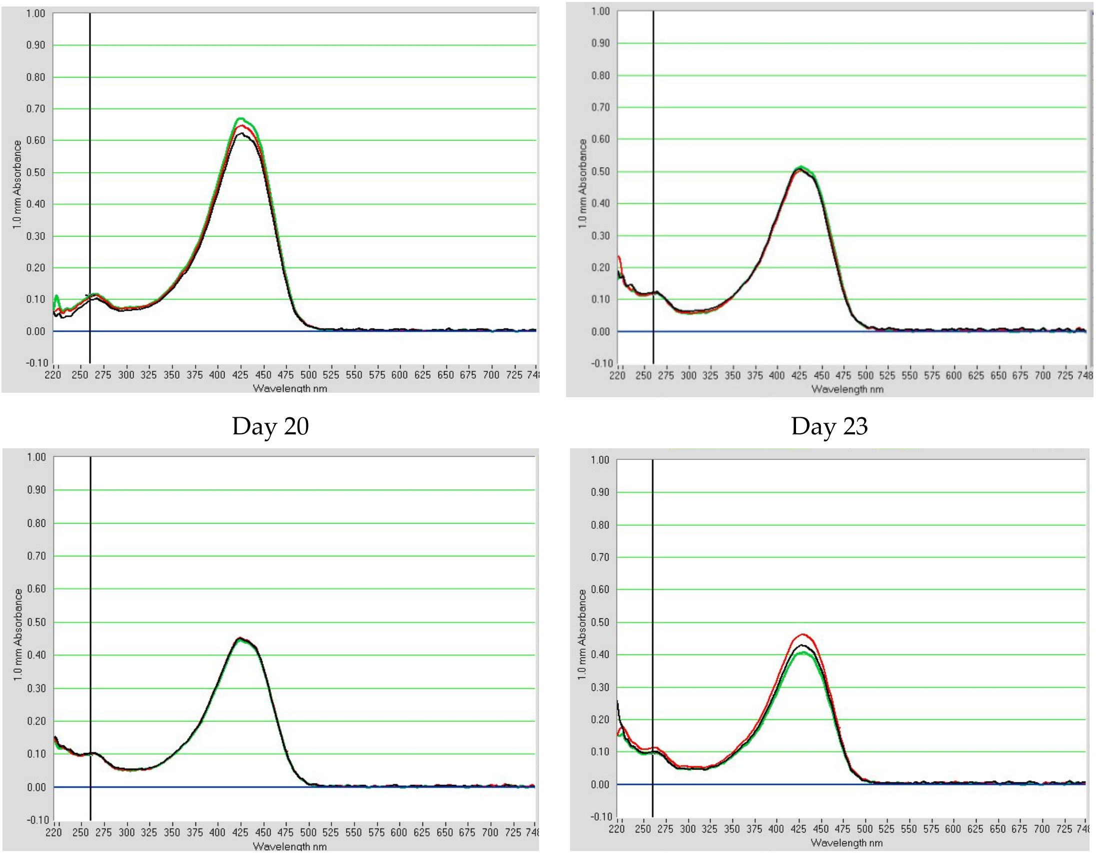

Figure S2. UV-VIS spectra recorded for FC degradation in the presence of air and light.

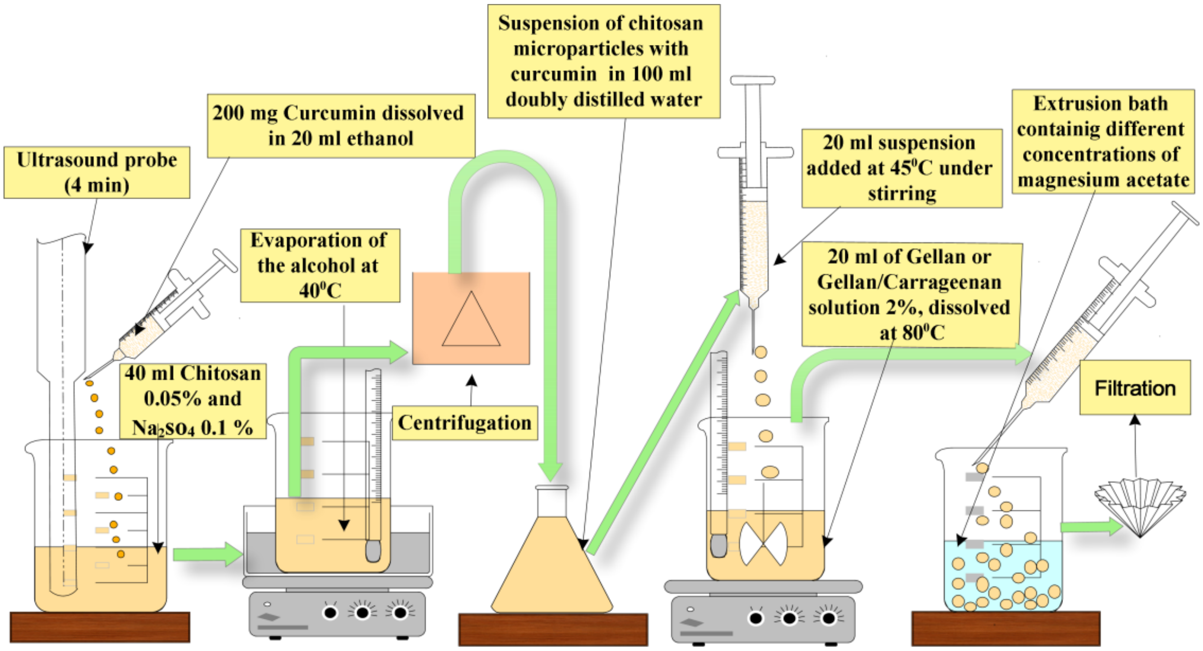

Figure S3. Schematic representation of the preparation process of obtaining the polysaccharides ComPs with immobilized curcumin.

Table S1. Variation of non-degraded curcumin (%) in time, in buffer solutions with different pH values, for the analyzed samples.

Sample	pH	Variation of the Non-Degraded Curcumin (%) in Time								k	$t_{1/2}$ (h)
		1 h	2 h	3 h	4 h	5 h	6 h	7 h	8 h		
FC	3	75.9 ± 1.9	72.8 ± 1	71.8 ± 0.9	70.7 ± 1.5	55.9 ± 2.9	53.3 ± 2.6	50.2 ± 2.7	47.7 ± 2.2	0.1 ± 0.005	6.84 ± 0.3
	6.8	76.6 ± 7.9	68.3 ± 5.8	62.4 ± 3.8	59.1 ± 2.2	56.6 ± 1.7	53.3 ± 3.8	49.1 ± 3.8	43.3 ± 1.7	0.11 ± 0.006	6.2 ± 0.3
	7.4	76.6 ± 2.9	66.6 ± 5.5	61.6 ± 1.7	57.5 ± 1.4	52.5 ± 3.6	50.8 ± 4.4	48.3 ± 3.8	40.8 ± 2.2	0.12 ± 0.001	5.7 ± 0.05
	9	60.8 ± 2.5	40 ± 4.3	30.81 ± 4.6	26.6 ± 3.6	20.8 ± 3.8	15.8 ± 0.8	12.5 ± 2.5	8.3 ± 1.4	0.32 ± 0.025	2.1 ± 0.1
P2C	3	92.9 ± 1.1	85.7 ± 0.7	82.8 ± 1.1	82.8 ± 1.1	77.1 ± 1.1	75.7 ± 1.9	69.9 ± 1.5	65.8 ± 1.3	0.051 ± 0.003	13.5 ± 0.8
	6.8	89.2 ± 1.2	85.7 ± 2	85.7 ± 2	76.1 ± 1.8	74.2 ± 1.6	74.2 ± 1.6	74.2 ± 1.6	71.3 ± 1.8	0.05 ± 0.002	13.6 ± 0.5
	7.4	94.3 ± 1.3	85.1 ± 0.7	84.1 ± 2.6	80 ± 1.3	70.3 ± 2	65.8 ± 0.7	62.9 ± 0.9	62.9 ± 0.9	0.053 ± 0.002	13.1 ± 0.6
	9	99.8 ± 0.9	99.8 ± 1.8	77.7 ± 2	74.2 ± 2.4	73.8 ± 2.2	70.9 ± 0.7	68.5 ± 1.8	65.8 ± 2.6	0.059 ± 0.001	11.8 ± 0.3
P4C	3	87.3 ± 1.4	80.4 ± 3	80.1 ± 1.4	69.8 ± 2.5	68.4 ± 3.6	65.8 ± 2.4	65.2 ± 2.1	64 ± 1.8	0.067 ± 0.003	10.35 ± 0.5
	6.8	88.6 ± 0.7	85.5 ± 0.15	78.6 ± 0.5	78.2 ± 0.5	66.2 ± 0.7	64.7 ± 0.3	63.2 ± 0.4	61.7 ± 0.1	0.067 ± 0.001	10.34 ± 0.1
	7.4	79.8 ± 0.5	69 ± 2.7	77.8 ± 1.3	70.8 ± 4.2	69.3 ± 2.1	69.2 ± 1.9	69 ± 0.5	62.5 ± 3.7	0.065 ± 0.001	10.7 ± 0.2
	9	77.3 ± 0.5	78.4 ± 3.3	75.4 ± 1.3	68.7 ± 4.1	67.2 ± 2.0	67.1 ± 1.8	66.8 ± 0.4	60.6 ± 3.5	0.071 ± 0.002	9.7 ± 0.3

Table S2. The TPC or TFC determined from free curcumin (FC) and curcumin extracted from P2C, P4C, and P5C samples irradiated or not irradiated with UVA. The results are expressed as mean values ± STDEV.

Sample	FC (Control)	P2C	P4C	P5C	FCUV (Control)	P2CUV	P4CUV	P5CUV
TPC, mg GAE/g particles	34.3 ± 0.6	33.3 ±1.3	31.4 ± 1.1	32.0 ± 1.3	39.0 ± 0.7	33.4 ± 0.4	31.8 ± 0.8	32.2 ± 0.3
TFC, mg CE/g particles	41.1 ± 1.2	34.3 ± 1.3	33.3 ± 1.8	34.1 ± 0.7	45.9 ± 1.2	35.5 ± 1.3	34.4 ± 0.7	35.3 ± 0.9
